# Reduced resting state connectivity and gray matter volume correlate with cognitive impairment in minimal hepatic encephalopathy

**DOI:** 10.1371/journal.pone.0186463

**Published:** 2017-10-12

**Authors:** Raquel García-García, Álvaro Javier Cruz-Gómez, Alba Mangas-Losada, Amparo Urios, Cristina Forn, Desamparados Escudero-García, Elena Kosenko, Juan Fermín Ordoño, Joan Tosca, Remedios Giner-Durán, Miguel Angel Serra, César Avila, Vicente Belloch, Vicente Felipo, Carmina Montoliu

**Affiliations:** 1 Centro Investigación Príncipe Felipe. Valencia, Spain; 2 Departamento Psicologia Basica, Clinica y Psicobiologia. Universitat Jaume I. Castellon, Castellón de la Plana, Spain; 3 Fundacion Investigacion Hospital Clinico Valencia. INCLIVA. Valencia, Spain; 4 Unidad de Digestivo-Hospital Clínico. Departamento Medicina, Universidad Valencia, Valencia, Spain; 5 Institute of Theoretical and Experimental Biophysics, Pushchino, Russia; 6 Servicio Neurofisiología, Hospital Arnau de Vilanova, Valencia, Spain; 7 Psychopatology and Neurophysiology Unit, Paterna Mental Health Center, CIBERSAM, Valencia, Spain; 8 Servicio Digestivo. Hospital Arnau Vilanova, Valencia, Spain; 9 ERESA, Unidad de RM, Valencia, Spain; 10 Departamento Patología, Facultad Medicina, Universidad Valencia, Valencia, Spain; Banner Alzheimer's Institute, UNITED STATES

## Abstract

**Background and aims:**

Minimal hepatic encephalopathy (MHE) is associated with cognitive alterations and changes in connectivity. We assessed the relationship of the abnormalities of resting-state functional connectivity (rs-FC) and gray matter (GM) volume with different cognitive alterations and biochemical parameters associated to MHE.

**Methods:**

Thirty-nine cirrhotic patients (26 without and 13 with MHE) and 24 controls were widely cognitive assessed with a battery of psychometric tests. Atrophy was determined using Voxel-Based Morphometry and rs-FC was assessed by independent component analysis. Receiver operating characteristic (ROC) curves was performed to assess the diagnostic utility of rs-FC and GM reduction for the discrimination of patients with and without MHE. Blood ammonia, cGMP, and levels of pro-inflammatory interleukins were measured.

**Results:**

MHE patients showed significant decrease of GM volume and lesser degree of rs-FC in different networks related to attention and executive functions as compared to controls and patients without MHE. There is a progressive reduction in rs-FC in the default mode network with the progression of cognitive impairment. MHE patients showed GM reduction in the right frontal lobe, right insula and right cerebellum compared to patients without MHE. Alterations in GM volume and rs-FC correlated with the scores of different cognitive tests.

**Conclusions:**

Decreased cognitive performance is associated by reduced rs-FC and GM atrophy in MHE patients. These changes could have predictive value for detecting MHE.

## Introduction

Hepatic Encephalopathy (HE) is a complex neuropsychiatric syndrome characterized by a functional alteration of the central nervous system associated to liver disease [1, 2]. Around 33–50% of cirrhotic patients without clinical symptoms of HE show minimal hepatic encephalopathy (MHE) with mild cognitive impairment, attention deficits [3,4] and impaired visuo-motor and bimanual coordination [5, 6]. MHE decreases the quality of life, and increases the probability of suffering HE [7, 8]. The "gold standard" for the MHE is the Psychometric Hepatic Encephalopathy Score (PHES) [1, 9]. However PHES is not sensitive enough to detect early neurological alterations, which are different for different patients [10].

Altered synchronization of neuronal activity between different areas may contribute to cognitive alterations typically impaired in MHE, such as attention and motor coordination [11]. Patients with MHE show white matter alterations related to cognitive impairment [12]. This could alter the functional connectivity (FC) of neural networks, causing neurological deficits. Moreover, a significant decrease in gray matter (GM) density in several brain areas has been described in cirrhotic patients [13]. MHE patients present focal damage in the precuneus (involved in the Default Mode network, DMN), which correlates with cognitive impairment [14]. This suggests that alterations in the integrity of the GM may be, at least in part, responsible for the alterations in FC.

Several fMRI studies indicate abnormal brain resting state functional connectivity (rs-FC) in cirrhotic patients with MHE, in the DMN and in some active neuronal networks, which could play an important role in cognitive dysfunction in MHE [15–19].

Resting state studies in MHE have focused on certain networks because they have functional characteristics involved in some of the cognitive domains altered in MHE [15–19]. However none of these studies use specific tests for the different cognitive alterations present in MHE.

In this study we aimed to assess the rs-FC of the brain globally, using the Independent Component Analysis (ICA) method and a large battery of psychometric tests, which assess different neurological functions: attention, concentration, mental processing speed, working memory and bimanual and visuomotor coordination, in order to establish new relationships between rs-FC and the different cognitive alterations associated to MHE.

We also assessed the relationship between alterations in GM integrity and rs-FC in different Resting State networks (RSNs) with some biochemical parameters that are associated to cognitive impairment in MHE.

The analysis of the GM integrity and rs-FC by fMRI would allow an earlier and more sensible detection of the MHE and to advance in the knowledge of the mechanisms responsible for the neurological deterioration associated to MHE. Moreover, it could help to characterize brain alterations occurring even before cognitive impairment is detected, which would allow addressing the patient's treatment earlier and improving the patient's quality of life as soon as possible.

## Patients and methods

### Participants

Forty-two patients with liver cirrhosis and 25 healthy controls without liver disease were enrolled into the study after written informed consent. After performing MRI four subjects (1 control and 3 patients) were excluded for posterior statistical analyses due to excessive head movement (translation > 2.5 mm or rotation > 2.5°), reducing the groups to 24 controls, 26 patients without MHE (nMHE) and 13 with MHE (Table 1). Patients were recruited between July 2015 and December 2016 from the outpatient clinics at Hospital Clinico Universitario and Hospital Arnau de Vilanova, in Valencia, Spain, and were included if they had clinical, biochemical, and histological evidence of liver cirrhosis. For controls, liver disease was discarded by clinical, analytical, and serologic analysis. All subjects were volunteers. Exclusion criteria were overt HE or history of overt HE, recent (<6 months) alcohol intake, infection, recent (<6 weeks) antibiotic use or gastrointestinal bleeding, use of drugs affecting cognitive function, hepatocellular carcinoma, or neurological or psychiatric disorder. Psychometric tests, attention and coordination tests (see S1 Supplementary methods) and blood collection were carried out on the same day. Cerebral fMRI was performed in the following week after neuropsychological assessment. Patients were classified as 26 without MHE and 13 with MHE according to PHES score (see S1 Supplementary methods).

**Table 1:** Composition of the different groups and etiology of liver disease.

	Controls	nMHE patients	MHE patients
**Total individuals**	24	26	13
**Gender (M/F)**	16/8	20/6	12/1
**Age** *	61 ± 6 (50–73)	63 ± 9 (46–81)	64 ± 11 (49–85)
**Alcohol**	-----	11	6
**HBV/ HCV/ Alcohol +HBV**	-----	0/11/1	0/3/1
**Others**		3	3
**Child Pugh A/B/C**	-----	23/3/0	8/5/0
MELD*	-----	9 ± 2	12 ± 5

*Values are expressed as mean ± SD. In brackets: age range. MHE, minimal hepatic encephalopathy; HBV, hepatitis B virus; HCV, hepatitis C virus. MELD, model end stage liver disease. The Child Pugh Score is derived from a score of 1–3 given for severity of ascites, hepatic encephalopathy, INR, albumin and bilirubin. The higher the score is, the more severe the liver disease.

Study protocols were approved by Scientific and Ethical Committees of Hospitals Clinico and Arnau Vilanova, Valencia, Spain, and conform to ethical guidelines of Helsinki Declaration. Composition of groups, age, and etiology of disease are in Table 1.

### MRI acquisition

All subjects underwent an MRI scan using a 3 T Philips Achieva scanner (Philips Medical Systems, Netherlands). Sagittal high-resolution three-dimensional 3D MPRAGE T1 images was acquired (TR = 8.42 ms, TE = 3.8 ms, matrix = 320 x 320 x 250, voxel size = 1 x 1 x 1 mm, flip angle = 8°). In addition, functional MRI resting-state data was acquired using a gradient-echo T2*-weighted echo-planar imaging (EPI) sequence (5 min, 150 volumes, TR = 2000ms, TE = 30 ms, matrix = 80 x 80 x 31, voxel size = 3 x 3 x 3 mm, flip angle = 85°). During the resting sequence, participants were instructed to remain motionless and to relax with their eyes open, to not fall asleep and to think of nothing in particular.

Data preprocessing are detailed in S1 Supplementary methods.

### Independent component analysis

Independent component analysis (ICA) was performed using the Group ICA of FMRI Toolbox (http://mialab.mrn.org/software/gift, version 3.0a); to find the predefined RSNs. Group-level spatial ICA was applied using the minimum description length criteria to determine the optimal number of Independent Components (ICs), and using the Infomax ICA algorithm to extract 30 ICs. Fifty iterations of ICA were performed using ICASSO to determine the reliability or stability of the ICA algorithm [20], and the estimated centrotypes were used as representative ICs. The individual IC maps and time courses were computed using back-reconstruction based on aggregate components of the ICA and the results from the data reduction step and then converted to Z scores. Finally, six networks related to cognitive processes were selected: the default mode network (DMN), the left fronto-parietal network (LFPN), the dorsal attention network (DAN), the sensory motor network (SMN), the salience network (SN) and the basal ganglia network (BGN).

### Statistical analysis

Regional GM volume and RSNs FC differences between three groups were assessed using ANCOVA design in SPM12 (Wellcome Trust Centre for Neuroimaging, UCL, London, UK). All the results were assessed at p<0.05 FWE cluster-corrected for the multiple comparisons in a combination with a threshold of p<0.001 at the uncorrected voxel level including gender as a nuisance variable. GM volume and eigenvalues of significant clusters derived from the previous ANCOVAS contrasts were extracted and included in correlation analyses, and in analysis of receiver operating characteristic (ROC) curves to assess the diagnostic utility of rs-FC in several networks and of GM volume for the discrimination of patients with and without MHE. Significant differences between groups in demographic, clinical and neuropsychological variables were assessed using ANOVA with Bonferroni post-hoc comparisons. Statistical significance was set at p<0.05. For these calculations, data were processed with the software package SPSS Version 20.0 (SPSS, Chicago, IL) and GraphPad Prism 6.0.

## Results

### Performance in neuropsychological tests

MHE patients showed reduced performance in all subtests from PHES, compared to controls and to patients without MHE. Patients without MHE got lower scores than controls in digit symbol test (DST), serial dotting test (SDT) and line tracing test (LTT) subtests from PHES.

Stroop, d2 test and bimanual and visuo-motor coordination were impaired in patients with MHE as compared to patients without MHE and control group.

Mental processing speed, measured by the oral Symbol digit modalities test (SDMT) and DST from PHES was altered in both groups of patients compared to controls, but patients with MHE showed a poor performance of these tasks when compared with patients without MHE. Finally, both group of patients also showed impaired performance in Digit Span and Letter-Number Sequencing tests. See Table 2 for specific results.

**Table 2:** Performance in neuropsychological tests and results of biochemical determinations.

	Controls	nMHE patients *P* vs. Control	MHE patients *P* vs. control	MHE patients *P* vs. nMHE	Global ANOVA *P* Values
***Neuropsychological tests***					
**PHES Global score**	0.9 ± 0.2	-0.9 ± 0.3***	-7.3 ± 1***	**p<0.001**	**<0.001**
**DST** (items completed)	44 ± 2	28 ± 2 ***	19 ± 2***	**p<0.01**	**<0.001**
**NCT-A** (seconds)	29 ± 2	39 ± 3	67 ± 11***	**p<0.001**	**<0.001**
**NCT-B** (seconds)	72 ± 5	109 ± 9	243 ± 46***	**p<0.001**	**<0.001**
**SD** (seconds)	59 ± 3	80 ± 3***	125 ± 10***	**p<0.001**	**<0.001**
**LTT** (seconds+errors)	98 ± 3	124 ± 6**	220 ± 16***	**p<0.001**	**<0.001**
**Bimanual coordination** (min)	1.9 ± 0.04	2.2 ± 0.05*	3.3 ± 0.3***	**p<0.001**	**<0.001**
**Visuo-motor coordination** (min)	2.3 ± 0.07	2.8 ± 0.1*	4.4 ± 0.5***	**p<0.001**	**<0.001**
**d2 Test–*TR*** *Values*	426 ± 16	300 ± 16***	238 ± 37***	**<0.05**	**<0.001**
**d2 Test—*TOT*** *Values*	393 ± 92	276 ± 15***	194 ± 28***	**<0.05**	**<0.001**
**d2 Test—*CON*** *Values*	156 ± 7	101 ± 7***	57 ± 15***	**p<0.01**	**<0.001**
**d2 Test *TA*** *Values*	157 ± 7	108 ± 7***	71 ± 11***	**p<0.05**	**<0.001**
***Stroop—Congruent Task*** ^a^	117 ± 3	98 ± 3***	75 ± 4***	**p<0.001**	**<0.001**
***Stroop—Neutral*** Task ^a^	82 ± 3	74 ± 3*	55 ± 3***	**p<0.001**	**<0.001**
***Stroop -Incongruent Task*** ^a^	46 ± 2	39 ± 2**	28 ± 2***	**p<0.01**	**<0.001**
**Oral SDMT** (correct pairings)	51 ± 1	38 ± 2***	24 ± 3***	**p<0.001**	**<0.001**
***Digit Span- Forward*** ^**b**^	9.3 ± 0.5	7.6 ± 0.3***	7.0 ± 0.4***	**ns**	**<0.001**
***Digits Span- Backward*** ^**b**^	6.5 ± 0.6	4.8 ± 0.3**	3.8 ± 0.5**	**ns**	**<0.001**
***Digits Span -Total score ^b^***	16 ± 0.9	12 ± 0.4***	11 ± 0.7***	ns	**<0.001**
***Letter-Number Sequencing test*** ^**b**^	9.9 ± 0.5	7.2 ± 0.5***	5.9 ± 0.7***	**ns**	**<0.001**
***Biochemical determinations***					
**Blood ammonia (μM)**	9 ± 1	27 ± 5**	35 ± 7**	**ns**	**<0.001**
**Plasma cGMP (pmol/ml)**	4 ± 0.2	8 ±1**	12 ± 1***	**<0.01**	**<0.001**
**Serum IL-6 (pg/ml)**	1.5 ± 0.1	2.4 ±0.2**	4.2 ± 0.6***	**<0.001**	**<0.001**
**Serum IL-18 (pg/ml)**	140 ± 17	227 ±17**	305 ± 27***	**<0.05**	**<0.001**

Values are expressed as mean ± SEM. nMHE and MHE, patients without and with Minimal Hepatic Encephalopathy, respectively; PHES, Psychometric Hepatic Encephalopathy Score; DST, Digit Symbol Test; NCT-A, NCT-B: Number Connection Test A and B; SD, Serial Dotting Test; LTT, Line Tracing Test; d2 test: TR, Total number of characters processed; TOT, Total correctly processed; CON, Concentration performance; TA, Total right answers.

^a^ Stroop test: Congruent task: number of words read in 45 seconds; Neutral task: number of colours read in 45 seconds; Incongruent task: number of items completed in 45 seconds. Oral SDMT, Symbol digit modalities test (oral version).

^b^ Right answers. Differences between groups were analyzed using one-way ANOVA followed by post-hoc Bonferroni. Significant differences compared to controls are indicated by asterisks

*p<0.05

**p<0.01

***p<0.001.

### Biochemical parameters

MHE patients show increased levels of IL6, IL18 in serum and of cGMP in plasma as compared to patients without MHE and controls. Patients without MHE show higher levels of these parameters than controls. Blood ammonia was significantly increased in patients compared to controls but there was no significant difference between patients without and with MHE (Table 2).

### Patients with MHE show reduced GM volume compared to patients without MHE and controls

**Fig 1:**
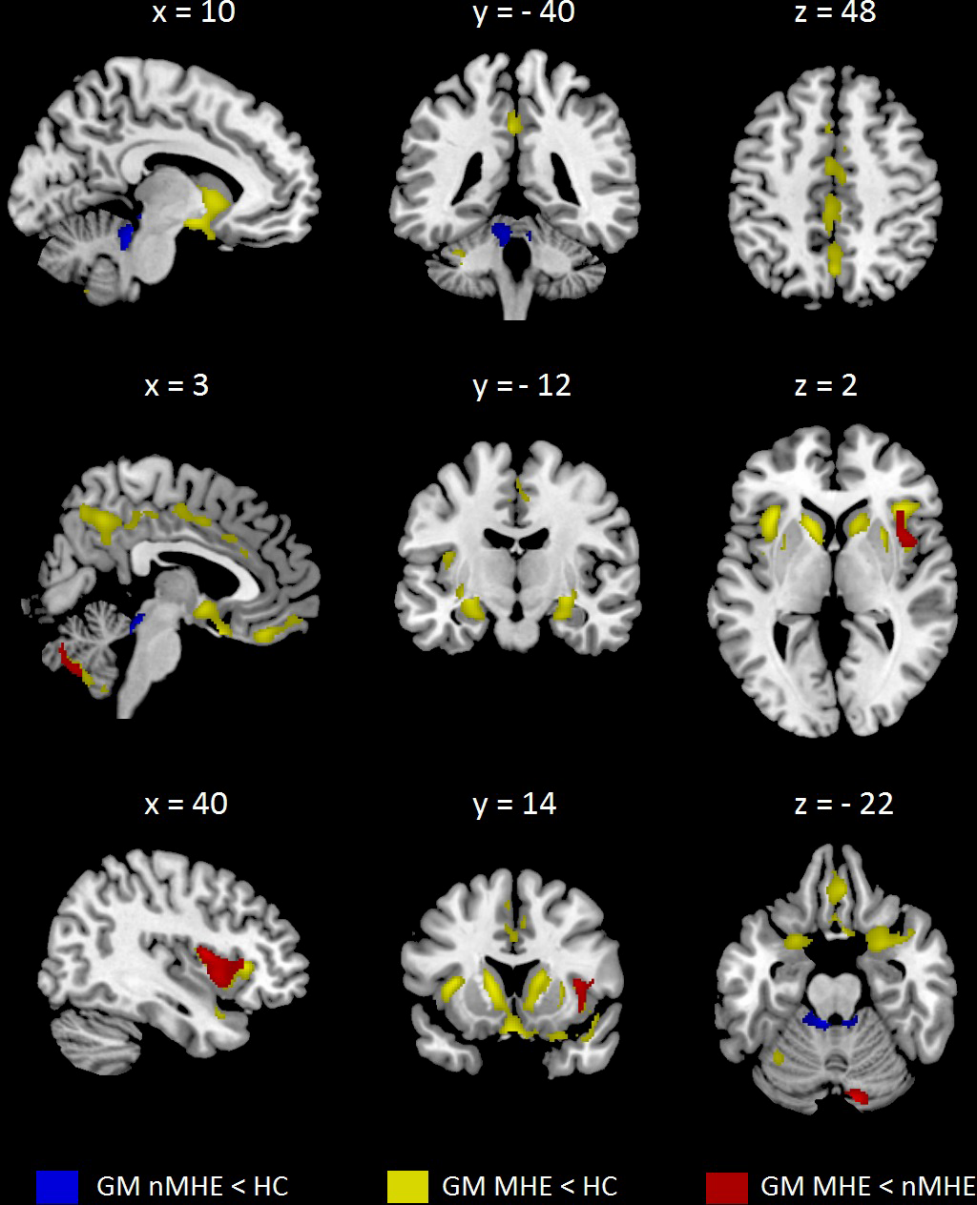
Localization of gray matter atrophy between groups including gender as nuisance covariate. All results were assessed at p<0.05 FWE cluster-corrected for the multiple comparisons in a combination with a threshold of p<0.001 at the uncorrected voxel level. HC, Healthy Controls; nMHE, patients without Minimal Hepatic Encephalopathy; MHE, patients with Minimal Hepatic Encephalopathy; GM, Gray Matter. Images are in neurological convention.

Both group of patients showed reduced GM volume as compared to healthy controls. Patients without MHE compared to controls showed GM atrophy focused in the bilateral cerebellum. As expected, MHE patients showed extended GM atrophy as compared to controls in several cortical and subcortical areas. Specifically, MHE patients showed reduction of GM in bilateral frontal areas (including the insula), bilateral precuneus, bilateral temporal pole and cerebellum. Moreover, GM atrophy in subcortical areas was focused in bilateral basal ganglia, bilateral hippocampus and parahippocampal gyrus, bilateral amygdala and bilateral cingulate cortex. For more specifically information regarding to GM atrophy in MHE patients see Table 3 and Fig 1.

**Table 3:** Areas showing significant gray matter (GM) volume differences between groups including gender as nuisance covariate.

Contrast	Anatomical Localization	k		MNI		t value
			x	y	z	
***HC > nMHE***	L Cerebellum (lobule III)	574	-8	-34	-16	6.36
	R Cerebellum (lobule III)		8	-37	-19	4.62
	L Cerebellum (lobule IV-V)		-13	-37	-19	4.37
***HC>MHE***	L Putamen	6444	-18	3	-12	5.73
	R Putamen		24	9	-5	5.05
	L Caudate		-14	15	5	5.24
	R Caudate		15	14	9	4.93
	L Insula		-34	21	3	5.27
	L Parahippocampal Gyrus		-20	-16	-21	3.42
	R Parahippocampal Gyrus		20	7	-22	3.61
	L Hippocampus		-25	-9	-15	4.30
	R Hippocampus		25	-9	-15	5.52
	L Amygdala		-21	-6	-15	3.69
	R Amygdala		25	5	-16	3.76
	L Rectus		-2	14	-20	5.02
	R Rectus		3	14	-21	4.90
	L Superior Temporal Pole		-26	8	-21	4.23
	R Superior Temporal Pole		26	8	-21	4.54
	L Olfactory Lobe		-8	7	-14	4.14
	R Olfactory Lobe		3	10	-14	3.51
	R Inferior Frontal Gyrus (pars orbitalis)		2	54	-14	3.52
	L Inferior Frontal Gyrus (pars triangularis)		-38	22	-2	4.36
	R Insula	920	39	3	11	5.32
	R Inferior Frontal Gyrus (pars triangularis)		44	25	3	4.44
	R Rolandic Operculum		45	-4	12	3.38
	L Rolandic Operculum	559	-38	-18	18	5.15
	L Heschl Gyrus		-36	-22	11	3.90
	L Middle Cingulate Cortex	1246	-3	-39	39	4.70
	R Middle Cingulate Cortex		2	-37	39	3.86
	L Posterior Cingulate Cortex		-2	-50	30	3.42
	R Posterior Cingulate Cortex		2	-50	30	3.63
	L Precuneus		-2	-63	47	4.39
	R Precuneus		3	-54	44	4.52
	L Paracentral Lobule		-5	-33	51	3.53
	L Medial Orbitofrontal Cortex	461	-2	60	-14	3.60
	R Medial Orbitofrontal Cortex		2	56	-14	3.54
	L Cerebellum (lobule VI)	433	-35	-44	-33	4.38
	L Cerebellum (Crus 1)		-35	-55	-29	4.14
	L Supplementary Motor Area	460	-2	-5	50	3.54
	R Supplementary Motor Area		2	-5	50	3.33
	L Cerebellum (lobule VII B)	491	-26	-73	-51	3.55
	L Cerebellum (lobule VIII)		-18	-69	-54	4.14
	L Cerebellum (lobule IX)		-6	-60	-50	3.08
	R Cerebellum (lobule IX)	323	5	-53	-54	3.83
	R Cerebellum (lobule VIII)		8	-67	-47	3.29
	Cerebellum (lobule VIII of Vermis)		-5	-69	-41	4.10
	L Anterior Cingulate Cortex	561	-3	33	20	4.01
	R Anterior Cingulate Cortex		2	32	20	3.55
	L Medial Frontal Gyrus		-2	22	41	3.42
***nMHE>MHE***	R Cerebellum (Crus I)	668	11	-81	-27	5.72
	R Cerebellum (Crus II)		24	-78	-39	3.81
	R Cerebellum (lobule VI)		14	-78	-22	3.92
	Cerebellum (lobule VII of Vermis)		6	-76	-27	3.94
	Cerebellum (lobule VIII of Vermis)		5	-69	-41	4.06
	R Insula	653	39	3	11	4.79
	R Rolandic Operculum		44	-2	14	3.54
	R Inferior Frontal Gyrus (pars opercularis)		45	14	8	3.38

All results were assessed at p<0.05 FWE cluster-corrected for the multiple comparisons in a combination with a threshold of p<0.001 at the uncorrected voxel level. HC, healthy controls; nMHE and MHE, patients without and with minimal hepatic encephalopathy, respectively. MNI: Montreal Neurological Institute; L, left; R, right; k: extend threshold k voxels; t: value of height threshold t.

Finally, MHE patients as compared to those without MHE showed reduction of GM in the right frontal lobe, right insula and right cerebellum (Table 3 and Fig 1).

Measurement of GM volume of bilateral insula and ganglia nuclei (caudate and putamen) individually, showed a significant GM atrophy in MHE patients compared to those without MHE and controls (see S1 Table).

### Relationship between GM atrophy and cognition

Loss of volume in right cerebellum correlated with the performance of PHES, especially with sub-tests assessing motor coordination: SDT and LTT. Further, GM volume in the right cerebellum also correlates with Stroop (incongruent task), visuomotor coordination, and d2 tests (Table 4).

**Table 4:** Correlations between GM volume and cognitive and biochemical variables.

Brain Region	Predictor	r	p
***Right Cerebellum***	PHES	0.657	<0.01
	Serial Dotting Test	-0.457	<0.01
	Line tracing test	-0.608	<0.01
	Stroop (Incongruent task)	0.426	<0.05
	Visuomotor coordination	-0.341	<0.05
	d2-TR	0.393	<0.05
	d2-TOT	0.424	<0.05
	cGMP	-0.396	<0.05
	IL-6	-0.650	<0.01
	MELD	-0.441	<0.01
	Child-Pugh	-0.472	<0.01
***Right Insula / Frontal Inferior Operculum***	PHES	0.755	<0.01
	Digit symbol test	0.481	<0.01
	NCT-A	-0.347	<0.05
	NCT-B	-0.512	<0.01
	Serial Dotting Test	-0.682	<0.01
	Line tracing test	-0.679	<0.01
	Stroop(Incongruent task)	0.499	<0.01
	d2-TR	0.385	<0.05
	d2-TOT	0.363	<0.05
	Oral SDMT	0.517	<0.01
	Digit Span -Backward	0.368	<0.05
	cGMP	-0.381	<0.05
	IL-6	-0.408	<0.05
	MELD	-0.444	<0.01
	Child-Pugh	-0.387	<0.05

The p and r values of the correlation analysis in all patients group are shown. Abbreviations: PHES, Psychometric Hepatic Encephalopathy Score; NCT-A, NCT-B: Number Connection Test A and B; d2 test: d2-TR, Total number of characters processed; d2-TOT, Total correctly processed; Oral SDMT, Symbol Digit Modalities test, oral version; MELD, model end stage liver disease.

On the other hand, GM volume in the right frontal lobe and right insula correlated with PHES score, incongruent task of Stroop test and oral SDMT test. The five subtests of PHES gave significant correlations with GM volume of this cluster. Weaker correlations were also found with d2 test and Digits span-backward (Table 4).

### GM reduction correlates with biochemical parameters associated to MHE

Increases in serum IL-6 and plasma cGMP levels showed a relationship with reduction of GM in the right cerebellum and in right insula, given the negative correlations found (Table 4). Finally, loss of volume in right cerebellum correlated with severity of liver disease, assessed by the Child Pugh and MELD scores. MELD score also correlated with loss of GM in right insula.

### Patients with MHE show decreased resting state functional connectivity

**Fig 2:**
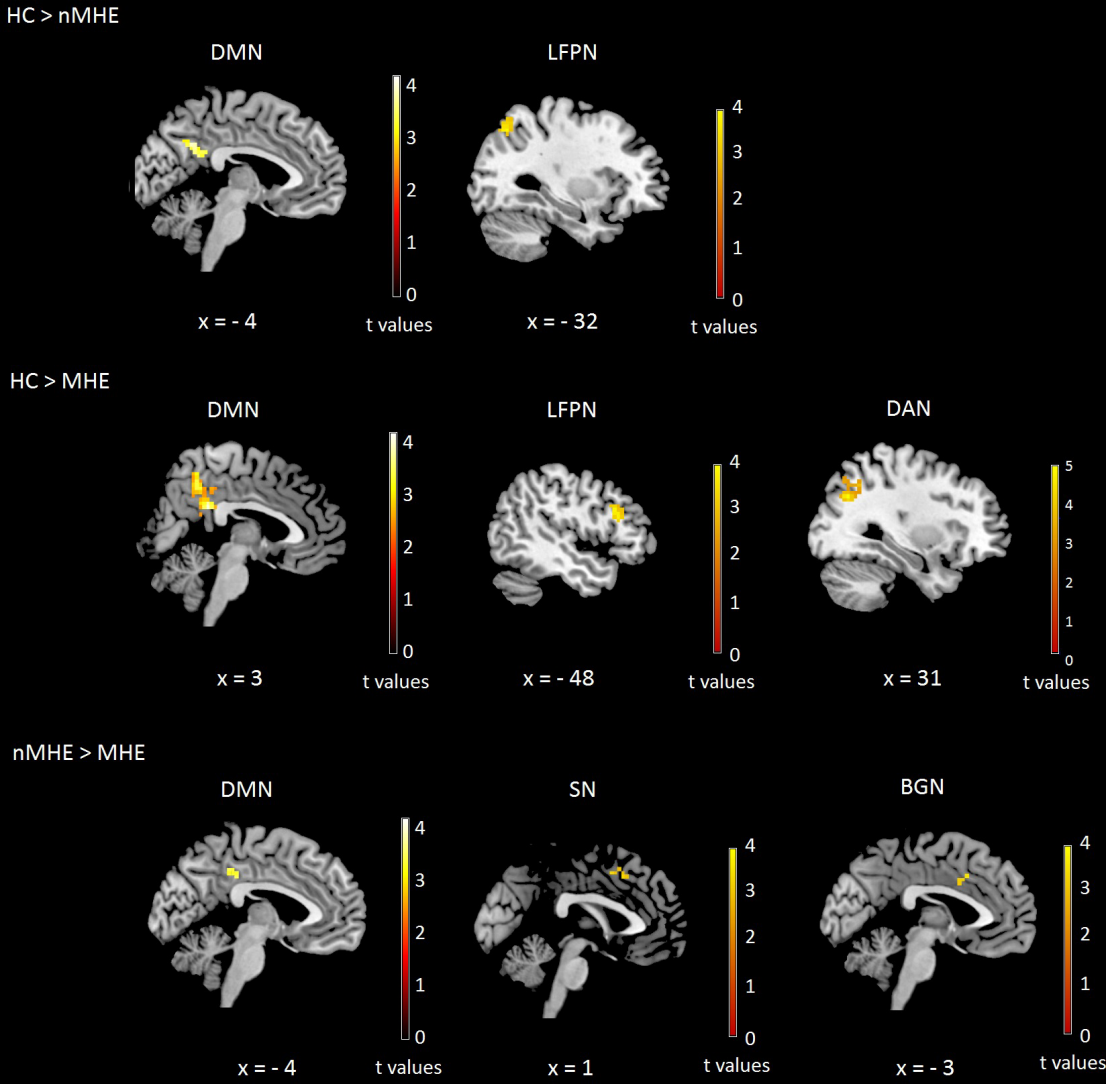
Differences among groups in the resting-state functional connectivity (rs-FC) networks including gender as a nuisance variable. All results were assessed at p<0.05 FWE cluster-corrected for the multiple comparisons in a combination with a threshold of p<0.001 at the uncorrected voxel level. HC, Healthy Controls; nMHE, patients without Minimal Hepatic Encephalopathy; MHE, patients with Minimal Hepatic Encephalopathy; DMN, Default Mode Network; LFPN, Left Fronto Parietal Network; DAN, Dorsal Attentional Network; SN, Salience Network; BGN, Basal Ganglia Network. Images are in neurological convention.

Results on the differences among groups in the rs-FC are presented in Table 5 and Fig 2. Within the DMN, patients with and without MHE showed a significantly decreased in rs-FC compared with controls in bilateral precuneus and bilateral posterior cingulate cortex. Further, patients with MHE also showed lower rs-FC than patients without MHE in the same area.

**Table 5:** Differences among groups in the resting-state functional connectivity (rs-FC) networks including gender as a nuisance variable.

	Anatomical Localization	k		MNI		t value
			x	y	z	
**Default Mode Network (DMN)**						
*HC > nMHE*	L Precuneus	39	-3	-57	33	3.65
	R Precuneus		3	-57	33	3.64
	L Cingulate Gyrus		-3	-48	24	3.95
	R Cingulate Gyrus		1	-49	30	3.94
*HC>MHE*	L Precuneus	114	-1	-47	42	3.72
	R Precuneus		6	-51	45	4.89
	L Cingulate Gyrus		-3	-45	27	4.14
	R Cingulate Gyrus		3	-45	27	4.67
*nMHE > MHE*	L Precuneus	23	-1	-48	42	3.66
	R Precuneus		3	-48	42	3.44
	L Cingulate Gyrus		-3	-39	42	3.51
	R Cingulate Gyrus		3	-39	42	3.96
**Left Fronto-Parietal Network (LFPN)**						
*HC > nMHE*	L Superior Parietal Lobule	41	-33	-66	48	4.61
	L Inferior Parietal Lobule		-30	-57	42	3.92
*HC > MHE*	L Inferior Frontal Gyrus	36	45	24	18	4.55
**Dorsal Attentional Network (DAN)**						
*HC > MHE*	R Middle Occipital Gyrus	47	30	-66	33	5.46
	R Superior Occipital Gyrus		27	-69	35	3.66
	R Angular Gyrus		30	-57	42	4.15
**Salience Network (SN)**						
*nMHE > MHE*	R Supplementary Motor Area	21	9	12	48	4.29
	L Supplementary Motor Area		-2	18	45	3.38
**Basal Ganglia Network (BGN)**						
*nMHE > MHE*	L Cingulate Gyrus	21	-6	12	36	4.09
	R Cingulate Gyrus		9	15	39	3.63

All results were assessed at p<0.05 FWE cluster-corrected for the multiple comparisons in a combination with a threshold of p<0.001 at the uncorrected voxel level. HC, healthy controls; nMHE and MHE, patients without and with minimal hepatic encephalopathy, respectively. MNI: Montreal Neurological Institute; L, left; R, right; k: extend threshold K voxels; t: value of height threshold t.

Within the LFPN, MHE patients showed a decreased connectivity in the left inferior frontal gyrus compared to controls, whereas patients without MHE presented lower connectivity than controls in the left superior and inferior parietal lobe. On the other hand, MHE patients showed a decreased rs-FC compared to patients without MHE in the SN, focused in the bilateral supplementary motor area and in bilateral anterior cingulate cortex of the basal ganglia network (Fig 2).

Patients with MHE showed a decreased connectivity compared to controls in some areas involved in the DAN, such as the right middle and superior occipital gyrus and the right angular gyrus.

Finally, no significant differences between groups were found for SMN.

### Correlations between rs-FC networks and cognitive and biochemical parameters

Results of the multiple regression analyses showed a significant (p<0.01) positive correlation in all patients group between the PHES score and rs-FC in the bilateral precuneus and cingulated gyrus of the DMN, bilateral supplementary motor area related to the SN, and bilateral anterior cingulate gyrus of the BGN. A good execution of Stroop test (incongruent task) correlated with a greater rs-FC in the bilateral anterior cingulate gyrus related to the BGN (Table 6).

**Table 6:** Correlations among resting-state functional connectivity (rs-FC) networks and cognitive and biochemical variables.

Group	Dependent variable	Predictor	r	*p*	Anatomical Localization
All patients	DMN	PHES	0.62	<0.01	Bilateral Precuneus and Cingulate Gyrus
All patients	DMN	Digit Symbol Test	0.38	<0.05	Bilateral Precuneus and Cingulate Gyrus
All patients	DMN	Serial Dotting test	-0.57	<0.01	Bilateral Precuneus and Cingulate Gyrus
All patients	DMN	Line tracing test	-0.54	<0.01	Bilateral Precuneus and Cingulate Gyrus
All patients	DMN	NCT-A	-0.35	<0.05	Bilateral Precuneus and Cingulate Gyrus
All patients	DMN	NCT-B	-0.43	<0.05	Bilateral Precuneus and Cingulate Gyrus
All patients	DMN	Bimanual coordination	-0.60	<0.01	Bilateral Precuneus and Cingulate Gyrus
All patients	DMN	Visuomotor coordination	-0.47	<0.01	Bilateral Precuneus and Cingulate Gyrus
nMHE	DMN	Oral SDMT	0.45	<0.05	Bilateral Precuneus and Cingulate Gyrus
All patients	SN	PHES	0.57	<0.01	Bilateral Supplementary Motor Area
All patients	SN	Serial Dotting test	-0.46	<0.01	Bilateral Supplementary Motor Area
All patients	SN	Line tracing test	-0.39	<0.05	Bilateral Supplementary Motor Area
All patients	SN	IL-6	-0.38	<0.05	Bilateral Supplementary Motor Area
MHE	DAN	IL-18	-0.78	<0.05	R Precuneus, R Parietal Lobe, R Occipital Middle and Superior Gyrus
All patients	BGN	PHES	0.48	<0.01	Bilateral Cingulate Gyrus
All patients	BGN	Serial Dotting test	-0.46	<0.01	Bilateral Cingulate Gyrus
All patients	BGN	Stroop test (Incongruent task)	0.51	<0.01	Bilateral Cingulate Gyrus
All patients	BGN	Child-Pugh	-0.44	<0.05	Bilateral anterior cingulate cortex

The p and r values of the correlation analysis are shown. Abbreviations: BGN, Basal Ganglia Network; DAN, Dorsal Attentional Network; DMN, default mode network; L, Left; LFPN, left frontoparietal network; nMHE and MHE, patients without and with minimal hepatic encephalopathy, respectively; MELD, model end stage liver disease; NCT-A, NCT-B: Number Connection Test A and B; Oral SDMT, Symbol Digit Modalities test, oral version; PHES, Psychometric Hepatic Encephalopathy Score; R, right; SN, Salience network.

Further, negative correlations are also showed in all patients between the execution of bimanual coordination and visuomotor coordination tests and LTT and SDT subtests from PHES with rs-FC in the precuneus and cingulate gyrus, related to the DMN. Finally, patients without MHE also showed a positive correlation between oral SDMT execution and rs-FC in bilateral precuneus and cingulate gyrus of DMN.

There were negative correlations between rs-FC in bilateral supplementary motor area within SN and visuo-motor coordination subtests from PHES (SDT and LTT tests), and also with the serum levels of the pro-inflammatory cytokine IL-6. The other cytokine tested, IL-18, showed negative correlation with rs-FC in DAN in the group of patients with MHE.

### Diagnostic accuracy of alterations in GM volume and functional connectivity in several RSNs for detection of MHE

We performed ROC curve analysis to assess the utility of alterations in GM volume and in rs-FC for the diagnosis of MHE. Given the good correlations of GM volumes in Right cerebellum and Right insula/Frontal Inferior Operculum clusters and PHES (Table 4), we performed a ROC curve analysis for the diagnosis of MHE. For this analysis we included GM volumes from these clusters resulting from the ANOVA contrast nMHE>MHE (eigenvalues). GM volume in right cerebellum showed an area under the ROC curve of 0.986 (confidence interval: 0.96–1; p<0.001) with 100% sensitivity and 91% specificity at a cutoff of 0.698 mm^3^. Right insula/Frontal Inferior Operculum cluster gave an area under the ROC curve of 0.983 (confidence interval: 0.95–1; p<0.001) with 92% sensitivity and 95% specificity at a cutoff of 0.566 mm^3^.

Moreover, we found that GM abnormalities in bilateral insula and basal ganglia nuclei could be predictive for MHE (see S2 Table).

The ROC curve analyses of rs-FC in DMN, SN and BGN for the diagnosis of MHE showed areas under the ROC curves of 0.96, 0.92 and 0.88, respectively (p<0.001) with excellent values of specificity and sensitivity (see S3 Table).

## Discussion

In this study we investigated the functional abnormalities of RSNs and GM atrophy in cirrhotic patients with and without MHE as compared to controls. Further, we assessed the relationship of these alterations with different cognitive alterations associated to MHE.

We found a significant decrease in rs-FC in MHE patients in attention-related networks such as the DMN, the LFPN, and the DAN, in the SN, involved in switching activity between DMN and the central executive network (CEN) and in the BGN. We also found a reduction in GM volume in brain areas involved in these networks, which could contribute to these alterations in rs-FC. Moreover, our results suggest that rs-FC changes within SN, BGN and DMN could have predictive value for detecting the MHE, and could be used as diagnostic biomarkers for MHE.

Changes in DMN have already been documented in MHE in previous studies [15–17], which show a progressive increase in DMN deterioration with the progression of cognitive impairment (negative correlations between sc-FC in some of the regions of DMN and the execution of some PHES subtests). Our results are in agreement with these studies, showing a reduction in rs-FC of DMN in patients without MHE compared to controls and a greater reduction of rs-FC in patients with MHE, as well as good correlations with the execution of the cognitive tasks. These results suggest a progressive alteration of the network with the advance of the disease, which support previous studies that consider HE as a continuum of neurocognitive dysfunction [21]. On the other hand, the fact that patients without MHE, although less affected than patients with MHE, also show alterations in rs-FC in DMN, could indicate that rs-FC is a more reliable index to elucidate early dysfunction induced by hepatic decompensation than neuropsychiatric evaluations. Patients without MHE showed lower rs-FC than controls in DMN, and LFPN, suggesting that alterations in rs-FC would be previous to volumetric alterations, and could explain the differences in execution of cognitive tests compared to controls.

We found alterations in the rs-FC in precuneus and cingulate gyrus, within DMN, which could be related to alterations in attention, working memory and executive control observed in the patients, all necessary for the correct execution of PHES. One task associated with the central precuneus is change of voluntary attention and working memory [22]. On the other hand, fMRI studies have suggested that abnormal activity in the prefrontal cortex and anterior cingulate cortex is associated with impairment of executive control of MHE [23]. Interestingly, Cheng et al [24] described a relationship between the normalization of amplitude of low-frequency fluctuation values in the precuneus after liver transplantation and the improvement of number connection test A performance.

The anterior precuneus is functionally connected to the motor cortex [25] and ventral anterior cingulate sends projections to the premotor cortex [26]. Alterations in the rs-Fc of precuneus and cingulate cortex with motor areas could explain the negative correlation between rs-FC and the performance of psychomotor tests in patients.

MHE patients showed reduced rs-FC compared to those without MHE in the SN, focused on the bilateral supplementary motor area. SN, composed of the bilateral anterior insular cortex and dorsal anterior cingulate cortex, is involved in the detection of and orientation towards external stimuli and internal events [27].

Volumetric data in this study show atrophy in the GM of the insula in MHE patients and also in bilateral supplementary motor area and anterior cingulate cortex, structural alterations that could explain the functional alterations and the lower connectivity of the SN. Insula is functionally connected to the supplementary motor area [28], which plays a crucial role in the planning of movements, and their initiation [29] and in bimanual coordination [30].

The significant GM reduction in insula in MHE patients could be affecting to the connectivity of this area with the bilateral supplementary motor area, thus leading to the reduced rs-FC of this cluster found in patients with MHE. Supplementary motor area shows a wide range of white matter connections with motor, language and limbic areas. These alterations, together with a widespread altered anatomical connectivity of white matter in the brain in MHE patients, as compared with controls or patients without MHE [12], could explain the disturbances in movement initiation involved in bradykinesia in MHE [31] and contribute to alterations in postural control in these patients [32]. Lower connectivity in this network could explain the low manual dexterity for the execution of PHES subtests assessing visuomotor coordination (SDT and LTT).

To date, few studies have shown structural and functional changes in the insula of patients with MHE compared to controls [19, 33]. We found a significant GM reduction in the bilateral insula in MHE patients compared to patients without MHE, which correlates with cognitive alterations.

Attention deficit is one of the earliest and key features in the development of MHE [3–5]. We also observed a significant decrease in rs-FC in MHE patients compared to controls in DAN, which agree with previous studies [15, 17]. DAN is responsible for the orientation of endogenous attention and associated with the deterioration of selective attention and is involved in the change in voluntary attention [34, 35].

The lower parietal lobe within LFPN is involved in a wide variety of cognitive functions, including working memory and focused attention [36]. The relationship between the normalization of amplitude values of low frequency fluctuations in the lower parietal lobe after liver transplantation and the improvement of cognitive functions in patients has been described [24]. In our study, although there were differences in rs-FC between controls and patients without and with MHE, no significant correlation was obtained with the performance of the psychometric tests.

Both DAN and LFPN networks are within the CEN, which is closely related to the SN. A”triple network” model has been recently proposed including the SN, the DMN and the CEN, in which SN would facilitate the change of activation between DMN and CEN, through appropriate transient signals that activate CEN to mediate cognitive control processes and deactivate DMN [27]. Functional coupling and anti-correlated matching between these three networks is critical for the execution of certain brain tasks such as working memory, attention and executive control [27, 37], all of them altered in MHE [3–5].

Aberrant intrinsic organization and interconnectivity of the SN, CEN and DMN is characteristic of many psychiatric and neurological disorders [27]. Chen et al [18] showed alterations in the connectivity of SN and its functional coupling with DMN and CEN in patients with MHE. Alterations in the SN and consequently in the activation and de-activation of CEN and DMN respectively could explain the attention and coordination deficits found in MHE patients.

We found a significantly decreased rs-FC in MHE patients compared to without MHE in left and right cingulate gyri within the BGN. Moreover, ganglia nuclei (caudate and putamen) showed a significant GM atrophy in MHE patients compared to those without MHE and controls. The BGN is involved in many neuronal pathways, including those associated with emotional, motivational, associative, and cognitive functions. A decreased connectivity between thalamus and basal ganglia in MHE compared to healthy controls has been reported [38].

Traditionally, the cerebellum has been considered as a regulating organ of motor function, but an association between cerebellar alteration and impaired executive function has been demonstrated in diseases with cognitive impairment [39, 40]. Interestingly, the cerebellar blood flow is increased in cirrhotic patients, and specifically elevated in the vermis of MHE group. These alterations in blood flow correlate negatively with the execution of the NCT-A and B of PHES [6]. Alterations in GM volume in cerebellum, along with alterations in blood flow, could explain the impairment in executive function and cognitive processing speed in patients evaluated through the performance of such PHES subtests.

The main factors contributing to cognitive impairment in HE are hyperammonemia and inflammation. A synergic effect of hyperammonemia and inflammation has been proposed to be the main responsible of the neurological alterations in HE [41]. We found that serum IL-6 levels correlate with SN connectivity in bilateral supplementary motor area, and also with a reduced GM volume in insula and in right cerebellum, suggesting that peripheral inflammation could be involved in alterations in visuo-motor coordination in MHE.

## Conclusions

In conclusion, our results suggest that rs-FC changes within SN, BGN and DMN are associated with cognitive alterations and could have predictive value for detecting the MHE, and could be used as diagnostic biomarkers for MHE. Moreover, GM volume in brain areas involved in these networks could contribute to these alterations in rs-FC and to cognitive alterations in MHE.

## Supporting information

S1 Supplementary methods Neuropsychological assessment; Biochemical determinations in blood; Data preprocessing. (DOC)

S1 Table Gray matter (GM) volume of basal ganglia nuclei and bilateral insula for each group. (DOC)

S2 Table Diagnostic accuracy of gray matter (GM) volume for detection of MHE. (DOC)

S3 Table Diagnostic accuracy of reduction in resting-state functional connectivity (rs-FC) for detection of MHE. (DOC)
