# Supplementary material for: Reduced resting state connectivity and gray matter volume correlate with cognitive impairment in minimal hepatic encephalopathy
Source: PLoS One. 2017 Oct 12;12(10):e0186463. doi: 10.1371/journal.pone.0186463 (PMC5638549; doi:10.1371/journal.pone.0186463)
Supplement: S1 Supplementary methods — (DOC) [file pone.0186463.s001.doc]

**Supplementary methods**

**Neuropsychological assessment**

MHE was diagnosed with Psychometric Hepatic Encephalopathy Score (PHES) [1,9] which includes five subtests: digit symbol test (DST), number connection test A and B (NCT-A and NCT-B), serial dotting test (SDT), and line tracing test (LTT). PHES score was calculated adjusting for age and education level using Spanish normality tables (www.redeh.org). Patients were classified as MHE when the score was ≤ −4 points [9].

Stroop test, bimanual and visuo-motor coordination tests, d2 test, Digit Span, Letter-Number sequencing, and oral Symbol digit modalities test (SDMT) were performed as in previous studies [5,10].

# **Biochemical determinations in blood**

Interleukins IL-6 and IL-18 were measured in serum using Human IL-6 and IL-18 Platinum ELISA kits from eBioscience (Bender MedSystems GmbH, Vienna, Austria). Blood ammonia was measured immediately after blood collection with the Ammonia Test Kit II for the PocketChemBA system (Arkay, Inc., Kyoto, Japan). Cyclic GMP (cGMP) in plasma was determined using the BIOTRAK cGMP enzyme immunoassay kit from Amersham (GE Healthcare, Life Sciences, UK).

**Data Preprocessing**

3d T1 images were reoriented to the AC-PC and pre-processed with an optimized VBM (Voxel-Based Morphometry) protocol using the Diffeomorphic Anatomical Registration Through Exponential Lie Algebra (DARTEL) included in SPM12 (Wellcome Trust Centre for Neuroimaging, London, UK). The pre-processing steps included image segmentation into GM, WM, and CFS tissues in a native space, spatial normalization to a population-specific template, modulation (to ensure the preservation of the overall amount of GM tissue) and finally smoothing using an isotropic Gaussian kernel of 8-mm full width at half maximum.

Resting-state fMRI scans were pre-processed using DPARSF Advanced tool v.4 *(Chao-Gan Y, Yu-Feng Z. DPARSF: A MATLAB Toolbox for ‘Pipeline’ Data Analysis of Resting-State fMRI. Frontiers in Systems Neuroscience. 2010;4:13)* and included: removing first 10 functional scans to allow for scanner calibration and adaptation of subjects to the scanning noise, slice timing correction for interleaved acquisition, realignment to the first scan of each session, head motion correction, co-register, spatial normalization to the Montreal Neurological Institute (MNI) space and spatial smoothing with an isotropic Gaussian kernel of 4-mm full width at half maximum (FWHM).

Four subjects -1 control and 3 patients (1 without and 2 with MHE) - were excluded for posterior statistical analyses due to excessive head movement (translation > 2.5 mm or rotation > 2.5°).
