# Supplementary material for: Reduced resting state connectivity and gray matter volume correlate with cognitive impairment in minimal hepatic encephalopathy
Source: PLoS One. 2017 Oct 12;12(10):e0186463. doi: 10.1371/journal.pone.0186463 (PMC5638549; doi:10.1371/journal.pone.0186463)
Supplement: S1 Table — (DOC) [file pone.0186463.s002.doc]

**S1 Table. Gray matter (GM) volume of basal ganglia nuclei and bilateral insula for each group**

| **Brain Area** | **Controls** | **Patients without MHE** | **Patients with MHE** | **ANOVA Global *P*** |
| --- | --- | --- | --- | --- |
| L Putamen | 4.75 ± 0.19 | 3.95 ± 0.25^*^ | 2.81 ± 0.33^***, a^ | <0.001 |
| R Putamen | 5.02 ± 0.2 | 4.46 ± 0.21 | 3.47 ± 0.32^***,a^ | <0.001 |
| L Caudate | 4.65 ± 0.14 | 4.27 ± 0.15 | 3.44 ± 0.25^***,b^ | <0.001 |
| R Caudate | 5.07 ± 0.09 | 4.59 ± 0.13^*^ | 4.01 ± 0.20^***,a^ | <0.001 |
| L Insula | 9.54 ± 0.14 | 9.24 ± 0.14 | 8.31 ± 0.24^***,b^ | <0.001 |
| R Insula | 8.78 ± 0.13 | 9.39 ± 0.16 | 7.60 ± 0.27^***,a^ | <0.001 |

Data are the mean ± SEM of GM volume for each group of subjects. Volume units are in mm^3^. Differences between groups were analyzed using one-way ANOVA followed by post-hoc Bonferroni. Significant differences compared to controls are indicated by asterisks: *p<0.05; **p<0.01; ***p<0.001; differences between patients with and without MHE are indicated by: ^a^p<0.05; ^b^p<0.01; MHE, Minimal Hepatic Encephalopathy; L, left, R, right.
