# Supplementary material for: Reduced resting state connectivity and gray matter volume correlate with cognitive impairment in minimal hepatic encephalopathy
Source: PLoS One. 2017 Oct 12;12(10):e0186463. doi: 10.1371/journal.pone.0186463 (PMC5638549; doi:10.1371/journal.pone.0186463)
Supplement: S2 Table — (DOC) [file pone.0186463.s003.doc]

**S2 Table. Diagnostic accuracy of gray matter (GM) volume for detection of MHE.**

| **Brain region** | **AUROC**  **(95% CI)** | ***p* value** | **Cutoff (mm^3^)** | **Sensitivity (%)** | **Specificity (%)** |
| --- | --- | --- | --- | --- | --- |
| R insula | 0.75 (0.58– 0.93) | 0.01 | 8.2 | 77 | 73 |
| L insula | 0.81(0.66– 0.95) | 0.003 | 8.8 | 77 | 73 |
| R caudate | 0.73 (0.55– 0.91) | 0.02 | 4.5 | 77 | 59 |
| L caudate | 0.76 (0.57– 0.95) | 0.01 | 4.1 | 77 | 73 |
| R putamen | 0.77 (0.58– 0.95) | 0.009 | 3.7 | 77 | 86 |
| L putamen | 0.75 (0.58– 0.93) | 0.01 | 3.4 | 77 | 82 |

AUROC, area under the receiver operating curve; CI, confidence interval. L, left; R, Right.
