# Supplementary material for: Reduced resting state connectivity and gray matter volume correlate with cognitive impairment in minimal hepatic encephalopathy
Source: PLoS One. 2017 Oct 12;12(10):e0186463. doi: 10.1371/journal.pone.0186463 (PMC5638549; doi:10.1371/journal.pone.0186463)
Supplement: S3 Table — (DOC) [file pone.0186463.s004.doc]

**S3 Table.**  **Diagnostic accuracy of reduction in resting-state functional connectivity (rs-FC) for detection of MHE.**

| **Brain Network** | **AUROC**  **(95% CI)** | ***p* value** | **Cutoff ^a^** | **Sensitivity (%)** | **Specificity (%)** |
| --- | --- | --- | --- | --- | --- |
| **DMN** | 0.96 (0.89-1) | <0.001 | 2.03 | 91 | 86 |
| **SN** | 0.92 (0.81-1) | <0.001 | 0.55 | 82 | 100 |
| **BGN** | 0.88 (0.76-1) | <0.001 | 1.06 | 82 | 81 |

^a^ Cutoff values are the difference in rs-FC between patients with and without MHE, expressed as the eigenvalues of significant clusters derived from the previous ANCOVAS contrasts. AUROC, area under the receiver operating curve; BGN, basal ganglia network CI, confidence interval, DMN, Default mode network, SN, salience network
